# Andersen-Tawil Syndrome

**Published:** 2006-01-01

**Authors:** Andrew H Smith, Frank A Fish, Prince J Kannankeril

**Affiliations:** The Department of Pediatrics, Vanderbilt University School of Medicine, Nashville, TN

**Keywords:** Arrhythmias, Genetics, ion channels, Andersen-Tawil syndrome

## Abstract

Andersen-Tawil syndrome (ATS) is a rare condition consisting of ventricular arrhythmias, periodic paralysis, and dysmorphic features.  In 2001, mutations in KCNJ2, which encodes the a subunit of the potassium channel Kir2.1, were identified in patients with ATS.  To date, KCNJ2 is the only gene implicated in ATS, accounting for approximately 60% of cases.  ATS is a unique channelopathy, and represents the first link between cardiac and skeletal muscle excitability.  The arrhythmias observed in ATS are distinctive; patients may be asymptomatic, or minimally symptomatic despite a high arrhythmia burden with frequent ventricular ectopy and bidirectional ventricular tachycardia.  However, patients remain at risk for life-threatening arrhythmias, including *torsades de pointes* and ventricular fibrillation, albeit less commonly than observed in other genetic arrhythmia syndromes.  The characteristic heterogeneity at both the genotypic and phenotypic levels contribute to the continued difficulties with appropriate diagnosis, risk stratification, and effective therapy.  The initial recognition of a syndromic association of clinically diverse symptoms, and the subsequent identification of the underlying molecular genetic basis of ATS has enhanced both clinical care, and our understanding of the critical function of Kir2.1 on skeletal muscle excitability and cardiac action potential.

## Introduction

Andersen-Tawil syndrome describes a rare condition consisting of ventricular arrhythmias, potassium-sensitive periodic paralysis, and developmental anomalies. [1,2] While two cases of familial periodic paralysis associated with ventricular ectopy were first described in 1963, [3] the suggestion of a potential syndromic association was not proposed until 1971, when Ellen Andersen and colleagues described the characteristic triad in an 8 year-old dysmorphic boy who experienced episodes of muscle weakness and ventricular extrasystoles. [4]  Nearly a quarter of a century later, 10 previously-described cases were reviewed, in addition to 4 new patients in 3 kindreds, who appeared to inherit the condition predominantly in an autosomal dominant pattern.  Noting for the first time the phenotypically heterogenous nature of this disease, this was also the first report to ascribe the name of Andersen’s syndrome to this constellation of symptoms. [1]  Subsequently, the syndrome was renamed Andersen-Tawil Syndrome (ATS) in recognition of the contributions of Dr. Rabi Tawil, who developed diagnostic criteria and refined understanding of this entity. [5]

Following its initial characterization, significant advances have improved our understanding of the genetic basis and molecular aspects of ATS.  In the seminal paper by Plaster et al, [2]  linkage analysis in a large kindred revealed a locus corresponding to a genetic region of over 40 cM on chromosome 17q23.  With the notion that both the periodic paralyses and abnormalities in myocardial cell repolarization were associated with ion channel mutations, so-called channelopathies, the 3 ion channel genes within the linked region (KCNJ2, CACNG1, and SCN4A) were suitable candidate genes.  SCN4A was known to underlie periodic paralysis without heart or developmental problems, [6] and the CACNG1 gene product was not detected in heart.  Due to the known function and expression pattern of Kir2.1, the protein encoded by KCNJ2, it was considered an excellent candidate gene for ATS.  Eight different mutations in KCNJ2 were initially identified in ATS patients, and functional characterization of 2 of the mutations revealed dominant negative effects on channel function.  These findings demonstrated that ATS mutations in KCNJ2, which encodes the Kir2.1 channel α subunit, appear to markedly impair its vital role in stabilizing resting membrane potential and mediating the terminal repolarization phase of the action potential, resulting in a predisposition to ventricular arrhythmias through mechanisms discussed below.

We will review the clinical manifestations of Andersen-Tawil syndrome, including distinguishing historical, physical examination, and electrophysiologic findings. We will discuss the genetic and molecular aspects of ATS, and will address the characteristic heterogeneity at both the genotypic and phenotypic levels.  Finally, we will explore various therapies devised to address the primary cause of morbidity and mortality in ATS, namely the propensity for life-threatening ventricular arrhythmias.

## Clinical Manifestations

Since the initial report of Andersen-Tawil syndrome, literature has emerged emphasizing the inconsistencies in phenotypic expression and disease severity [7]. Such variability has been described among kindreds, as well as among members of the same families [8].  This variability has made the diagnosis of ATS difficult.  In fact, half of the new patients described in the initial paper proposing the term “Andersen’s syndrome” were diagnosed only after repeated evaluations [1].  Unpredictability is seen not only in dysmorphic characteristics, but electrophysiologic manifestations as well.  Although ATS is inherited in an autosomal dominant pattern, given its heterogenous expressivity, a family history of associated characteristics may not be elicited.  In fact, there is up to a 20% incidence of non-penetrance in individuals with KCNJ2 mutations.  Additionally, up to 40% of patients with ATS do not have KCNJ2 mutations, further confounding the ability to utilize genetic screening as a diagnostic tool [5].

### Dysmorphic features

Appreciation of dysmorphic manifestations comprise one of the three fundamental elements in the evaluation of ATS.  Features initially described included short stature, hypertelorism (wide-set eyes), low-set ears, palatal defects, mandibular hypoplasia, single palmar crease, crytporchidism, and slight bilateral ptosis [4].  In a recent series of 36 KCNJ2 mutation carriers, 78% had at least two dysmorphic features, the most common being clinodactyly (permanent medial or lateral curvature of a finger or toe, seen in 64%) and mandibular hypoplasia (44%) [8]. Other features described have included scoliosis, hyperthyroidism, vaginal atresia, and unilateral dysplastic kidney; structural cardiovascular anomalies such as a bicuspid aortic valve with or without associated coarctation, and valvular pulmonic stenosis have been reported as well [9]. Also noted in one series was that the severity of facial dysmorphism did not correlate with the severity of cardiac or skeletal muscular involvement [10].

### Periodic Paralysis

Periodic paralysis serves as another key element to the clinical diagnosis of ATS.  Historically, the age of onset of weakness is highly variable, though typically within the first two decades of life [10]. These episodes typically will present before any cardiac symptoms, and are usually manifested following periods of prolonged physical exertion.  Examination reveals a muscular weakness that is proximal in nature, and may be associated with muscle wasting.  Attacks have been demonstrated in patients with varying serum potassium levels (either hypo-, hyper-, or normokalemia), though levels have been found to remain consistent among an individual kindred.2  Potassium challenges have been found to at times either induce or even successfully treat episodic weakness [1,10]. Noted in one kindred was a sex-specific expressivity, with periodic paralysis appearing to segregate as an autosomal dominant trait only among males [9]. Additional findings have included markedly elevated serum creatine kinase levels, normal electromyogram, and tubular aggregates or minimal mypoathic changes apparent on pathologic examination of skeletal muscle biopsy [1,8]. 

### Cardiac Manifestations

**Figure 1:**
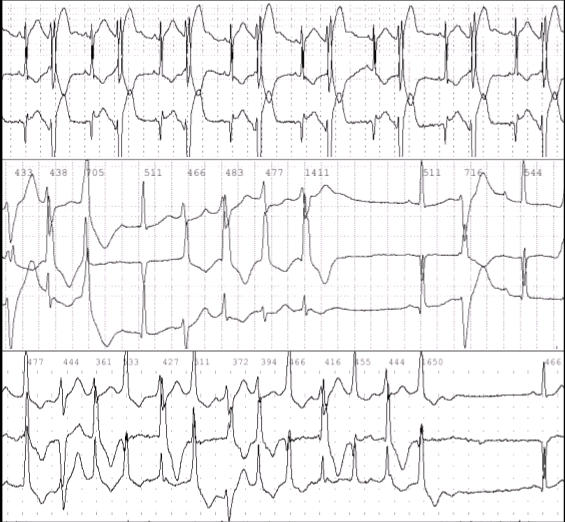
Ambulatory ECG tracings of ventricular arrhythmias in Andersen-Tawil Syndrome.  The top panel recorded in a newborn boy with the G144S mutation in KCNJ2 shows sinus rhythm with premature ventricular beats in a bigeminal pattern.  The middle and bottom panels recorded in the boy’s mother with the same mutation show runs of polymorphic and bidirectional ventricular tachycardia

True to its variability in phenotypic expression, there exists a wide range of electrophysiologic manifestations in ATS.  Some patients have QT interval prolongation, while others demonstrate normal QT intervals.  Prominent U waves in the anterior precordial leads are common in ATS [11]. Although U waves can be observed in normal individuals at low heart rates or in hypokalemic states, these are described in ATS patients at higher heart rates, suggesting that this may represent a manifestation of disease rather than a normal variant [8]. Associated arrhythmias range from isolated premature ventricular beats to complex ventricular ectopy and polymorphic ventricular tachycardias such as bidirectional ventricular tachycardia or less commonly *torsades de pointes* (see Figure 1) [2,11,12]. Complete right bundle branch block, left bundle branch block, bifasicular block, and first-degree atrioventricular block have also been described in patients with known KCNJ2 mutations [8,11]. Both nonfatal cardiac arrest with documented torsades de pointes, and sudden death, thought to be less common relative to its long QT syndrome (LQTS) counterparts, have also been reported [136]. Sex-specific expressivity of the arrhythmia phenotype has been described in 1 family, where 13 of 16 female carriers, but no male carriers, demonstrated a history of ventricular arrhythmias [9]. While gender differences in cardiac repolarization are well described but not completely understood,[14] hormonal modulation of arrhythmia risk is suggested by our observation that arrhythmias in ATS may be quiescent during pregnancy.

The association between ATS and a prolonged corrected QT interval has recently been called into question. Citing its relationship to other disorders of myocellular repolarization, predisposition to ventricular arrhythmias,  and the purported presence of a prolonged QT interval in 71% of all KCNJ2 mutation carriers, it had been suggested that ATS be classified as LQT7 [8]. A systematic assessment of ATS patient electrocardiograms was recently completed with conflicting results.  Specifically, in ATS patients with and without KCNJ2 mutations, median corrected QT intervals were minimally longer (20 ms) than controls, remaining within the normal range (median, 440 ms); in fact, only 17% of ATS patients in this series had a QTc of over 460 ms.  Additionally, it was noted that those patients possessing KCNJ2 mutations appeared to have a distinctive electrocardiographic phenotype, consisting of a prolonged terminal T wave downslope, a widened T-U wave junction, as well as biphasic and enlarged U waves; such findings were not evident in either ATS patients lacking KCNJ2 mutations or in healthy controls.  Accordingly, some now recommend that patients with KCNJ2 mutations be classified as ATS1, rather than LQT7 [11].

A well-characterized feature of ATS is its propensity for ventricular arrhythmias. In addition to isolated premature ventricular beats, ventricular tachycardias (VT) involving a beat-to-beat variability in axis (polymorphic ventricular tachycardia), such as bidirectional ventricular tachycardia and torsades de pointes, have been described [2,12,15]. *Torsades de pointes* and its potential for progression to life-threatening ventricular fibrillation is a well-known complication associated with the prolongation of the plateau phase of the action potential seen in congenital and acquired long QT syndromes [16,17].  Bidirectional ventricular tachycardia is a distinct form of polymorphic VT, characteristically associated with intracellular calcium overload and seen in catechecholaminergic polymorphic ventricular tachycardia (CPVT) and digitalis toxicity [12,18,19]. A relationship to intracellular calcium overload and Na+/Ca2+-mediated triggering of arrhythmic activity has been proposed in ATS [8].  Bidirectional VT, though rare, has been described extensively in association with ATS [2].  Indeed, bidirectional ventricular tachycardia in the absence of other causes should prompt a thorough search for other features of ATS in the patient and kindred.  In a cohort of 36 carriers of KCNJ2 mutations, a relatively low rate of syncope and cardiac arrest was described, despite a high arrhythmia burden.  Ventricular arrhythmias were noted in 88% of patients, with nonsustained VT present in 65% of probands, and bidirectional VT seen in 18% of probands.  Only 2 patients experienced nonfatal cardiac arrest (1 with documented *torsades de pointes*) and there were 4 episodes of syncope.  Additionally, there were no cases of sudden cardiac death, nor was there a reported family history of sudden death among subjects [8]. Interestingly, the incidence of symptoms such as syncope in this cohort was less than 20%, markedly lower than the 63% and 46% reported in LQT1 and LQT2 syndromes respectively [20].

As ATS is a rare disorder, there exists no literature currently which correlates particular genotypes or clinical features with an assessment of risk for development of life-threatening ventricular arrhythmias and sudden death.  While such reports of sudden death are rare in the literature, ATS patients remain predisposed to the development of polymorphic ventricular tachycardias such as bidirectional VT and torsades de pointes; with their potential for deterioration to ventricular fibrillation and hemodynamic collapse.  These patients consequently remain at an increased risk for sudden cardiac death relative to the general population. 

## Diagnosis

Given the heterogenous nature of the clinical manifestations of Andersen-Tawil syndrome, a definitive diagnosis at times is elusive.  Manifestation of at least two phenotypic classes (skeletal, cardiac, or developmental) has been thought to be necessary for diagnosis of ATS [8]. While some patients may manifest aspects consistent with all three classes of the clinical anomalies, others, (including those with known KCNJ2 mutations) may manifest only a single phenotypic classification.  In these instances, some authors have suggested diagnosing such patients should they have a substantial family history [5]. Noting that to date, about 60% of Andersen syndrome patients are found to have mutations in Kir2.1, screening for KCNJ2 mutations also provides an increasingly valuable tool in either confirming or establishing diagnosis in a phenotypically heterogenous condition.

## Molecular manifestations

### Kir2.1

Mutations in KCNJ2, which encodes the a-subunit of Kir 2.1, an inwardly-rectifying potassium channel, have been identified as the genetic defects underlying the clinical phenotype of Andersen-Tawil syndrome [2].  The Kir2 family of potassium subunits is expressed in both heart and skeletal muscle, and is the first disordered channel associated with phenotypic manifestations within both skeletal and cardiac muscle [21,22]. Kir2.1 subunits form a 427 amino acid protein, with 2 transmembrane domains and an extracellular pore forming loop containing the GYG amino acid sequence responsible for determining channel selectivity for potassium. The Kir2.1 channel functions as an inward rectifier, its conductance changing with voltage differences across the membrane. The term inward rectification indicates that while there is an inward flow of potassium current at hyperpolarized potentials, the same voltage gradient with depolarization produces much less outward potassium flow.  At potentials more negative to - 20 mV, the Kir channels increasingly permit potassium efflux, providing a current which mediates the terminal phase of repolarization.  The current provided by inward rectifiers such as the Kir2.1 channel, termed IK1, has consequently been shown to play a role in the determining cardiac excitability, and to “set” the resting potential [23,24].

### Kir2.1: Molecular pathology

Four individual Kir2.1 proteins co-assemble to form a functional tetrameric channel. While injection of normal Kir2.1 cRNA into oocytes results in potassium currents with strong inward rectification, injection of mutant (D71V and R218W) cRNA into oocytes resulted in no detectable current [2]. These results raise 2 possibilities: the mutant proteins either failed to co-assemble, or assemble normally but impair channel function. The latter was suggested by the finding of “dominant negative” suppression of channel function; > 50% reduction in current with coexpression of normal and mutant Kir2.1 subunits.  It is proposed that mutant proteins assemble with normal proteins into tetrameric channels, but the presence of 1 or more mutants in the channel is sufficient to disrupt current flow. Assuming random expression and co-assembly, only 1 out of every 16 channels would consist of 4 normal subunits, and function normally, while 15 of 16 would harbor at least 1 mutant protein.  Indeed, coexpression of the D71V mutant cRNA with normal cRNA resulted in a current approximately 1/16^th^ of normal current.

While other mutations have been described involving the external pore forming loop of Kir2.1,[7] mutations in KCNJ2 impairing channel interactions with phosphatidylinositol 4,5-bisphosphonate (PIP2) are thought to play a significant role as pathogenic mechanism in ATS.[25]  PIP2 is a membrane-bound second messenger which activates Kir2.1, among other inward-rectifiers, and promotes an open-channel position [5]. Also, in vitro it was demonstrated that in general (and especially with the previously described R218W mutation), these mutations resulted in impaired channel- PIP2 interactions, affecting whole-cell current and marked decreases in open-channel probability [25]. Consequently, impairments in PIP2 - mediated channel function would result in an increase in mean closed time, as has been described, resulting in a decrease in channel activity and manifestation of disease phenotype.

### Effects of impaired I_K1_

Decelerates  repolarization/prolongs action potential duration/depolarizes, destabilizes resting membrane potential

The effects of reduced I_K1_ in a theoretical model of rabbit ventricular myocyte demonstrated a prolongation of the terminal phase of the action potential [8]. Reductions in extracellular potassium concentrations along with decreases in I_K1_ were also associated with development of “terminal-phase” early after depolarizations (EADs), leading to spontaneous action potentials and providing a possible explanation for the frequent ventricular ectopy seen in patients with ATS. The term “terminal-phase” was used to distinguish the observed phenomena from the typical EAD’s seen in LQTS that arise from the plateau or early repolarization phase of the action potential.  Interestingly, also noted was the sodium-calcium exchanger-dependent development of delayed after depolarizations, much like those seen with digitalis toxicity. This may underlie the risk for bidirectional VT that is often observed in ATS.

Using adenoviral gene transfer techniques, Miake and colleagues were able to further delineate the in vivo role of I_K1_ in cardiac repolarization, studying isolated ventricular myocytes from adult guinea pig with both augmented and diminished I_K1_ expression [26]. “Dominant negative” reduction in I_K1_ was achieved by over expression of mutant Kir2.1with the GYG potassium selectivity motif replaced with AAA.  This resulted in prolongation of action potential duration (APD), deceleration of phase 3 repolarization, and depolarization of the resting membrane potential (RMP). Conversely, over expression of human Kir2.1 with a consequent increase in I_K1_ led to significant APD shortening with hyperpolarization of RMP and acceleration of phase 3 repolarization.

Clinical application of the recent understanding of the role of Kir2.1 and its corresponding current, I_K1_, continues to evolve. Patients with ATS and KCNJ2 mutations demonstrate a distinct change in U wave morphology, an electrocardiographic characteristic associated with frequent premature ventricular beats and nonsustained VT, potentially demonstrating an increased vulnerability to ventricular arrhythmia [11].  Mutation-induced reductions in I_K1_ have been proposed to lead to a prominent U wave morphology and ventricular ectopy through an increase in transmural dispersion of repolarization, leading to action potential duration changes that vary in degree from one region of myocardium to another, and potentially serving as the substrate for reentrant arrhythmias.  Interestingly, the gain-of-function mutations in KCNJ2 and resulting increases in I_K1_ have also recently been correlated with disease.  The resulting decreases in action potential durations leads to accelerated atrial and ventricular repolarization characteristic of the short QT syndrome.  In the form of short QT syndrome (SQT3) associated with a KCJN2 mutation, ventricular fibrillation has been observed [27].

### Molecular Genetics

ATS occurs sporadically, or is inherited in an autosomal dominant manner.  Mutations in KCNJ2 were the first described as responsible for the phenotype of ATS.  Kir2.1 subunits, encoded by KCNJ2, combine to form tetrameric channels with additional Kir2.1 subunits,[28] or with other subunits of the Kir2.x subfamily [29]. Mutations in KCNJ2 have been demonstrated to impair Kir2.1 channel function usually through a mechanism of dominant negative suppression [30]. The dominant-negative nature of ATS mutations has been well-described, whereby heterogenous expression of wild type Kir2.1 with any number of mutant Kir2.1 subunits results in a loss of current [2,8,30]. Also demonstrated was the ability of mutant Kir2.1 to exert dominant-negative suppressive effects upon other members of the Kir2.x subfamily, possibly serving as an explanation for the interfamilial phenotypic variations in expressivity [29].

Between 6-20 % of individuals with identified KCNJ2 mutations appear phenotypically normal (non-penetrant) [2,5,7-9]. Additionally, approximately 30-40% of patients with ATS do not harbor KCNJ2 mutations [5,8]. In the first demonstration of a relationship between KCJN2 mutations and ATSs, 3 of 16 families examined had no mutations in the coding regions of KCNJ2, with no apparent phenotypic differences in those with KCNJ2 mutations and those without [2].   Several mechanisms for this genotypic heterogeneity have been proposed, including mutations in KCNJ2 regulatory proteins, and mutations in other members of the Kir2.x subfamily, as these subunits assemble in heterotetramers to form functional channels [29].

## Treatment

Therapy for ATS is directed toward its phenotypic manifestations which lead to significant morbidity, namely associated episodic weakness and most importantly its association with life-threatening ventricular tachyarrhythmias.  From initial reports, therapies such as oral potassium supplementation, sodium restriction, spironolactone, and acetazolamide have anecdotally been shown to ameliorate symptoms of weakness [1,3,10,31]. We have obserevd improvement with oral contraceptive therapy, with exacerbation of weakness after discontinuation in a female patient with ATS. Unfortunately, perhaps due to the genotypic and phenotypic heterogeneity in disease, in concert with erratic and paradoxical exacerbation of symptoms with therapy, no therapeutic standards exist to date.

**Figure 2:**
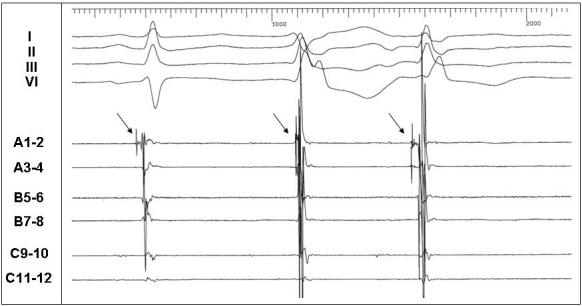
Intracardiac electrograms during spontaneous ventricular ectopy in a patient with Andersen-Tawil syndrome.  Displayed are surface leads I, II, III, V1, and intracardiac electrograms recorded from the anterior left ventricle.  The first sinus beat is followed by 2 successive ventricular ectopic beats, each with a different morphology.  The arrows indicate Purkinje potentials preceding the local ventricular electrograms in sinus rhythm and the ectopic beats.  The first ectopic beat is from an adjacent Purkinje site - hence the late Purkinje relative to the QRS.  (Figure courtesy of Dr. Prashanthan Sanders, Dr. Frederic Sacher, and Dr. Michel Haissaguerre, Hôpital Cardiologique du Haut-Lévêque, Bordeaux, France)

While the cardiac sequelae of ATS hold the greatest potential for morbidity and mortality, they remain the most problematic in devising effective medical therapeutic options.  Given the phenotypic heterogeneity that complicates diagnosis, this variability also plays a role as a complicating factor in effective risk stratification. The most concerning aspect of ATS, paroxysmal ventricular tachycardia leading to syncope or ventricular fibrillation with cardiac arrest, is only infrequently inducible with invasive electrophysiologic study [12]. Recently, radiofrequency catheter ablation targeting premature ventricular beats, which may serve as triggers for sustained ventricular arrhythmias, has been performed in other genetic arrhythmia syndromes, including LQTS and Brugada syndrome [32][33]. This may be an attractive approach in ATS due to the frequent ventricular ectopy.  Initial attempts to map ventricular ectopy in patients with ATS reveal that the ectopic beats originate from different parts of the left Purkinje network (personal communication, P. Sanders, 2005; see Figure 2).  Although to date, catheter ablation in ATS has not been reported, in a patient with arrhythmic storms, this approach could be considered.

Successful pharmacologic therapy remains elusive, lacking effectiveness in reducing the frequency of ventricular ectopy; [1] in fact, even from early reports such therapy has been recognized to exacerbate symptoms of weakness [1][3]. We have observed similar phenomena: improved rhythm control but worsening weakness, or improved muscle strength, but increased ventricular ectopy. Reports of antiarrhythmic drug efficacy remain anecdotal, and conflicting. While calcium channel blockers are theorized to address the proposed arrhythmogenic substrate of intracellular calcium overload, and have been shown to terminate bidirectional VT in ATS,[15] they have also been shown ineffective in reducing ventricular ectopy [12].  Similarly, amiodarone has been described as a successful therapy for VT in a patient with ATS due to the R218W KCNJ2 mutation [31]. The authors suggested  a possible pharmacogenetic link between the R218W mutation and treatment response.  However, subsequently amiodarone was implicated in inducing *torsades de pointes* in a different ATS patient who also harbored the R218W mutation [34]. While amiodarone may exacerbate early afterdepolarizations and predispose to TdP with its inhibition of I_K1_ and I_Kr_, its effects in a given patient may not be predictable.  Pacing in combination with nicorandil was successful in treating ventricular arrhythmias in the latter patient.  Pacing, by increasing heart rate and nicorandil, by increasing outward potassium current through I_KATP_, resulted in attenuation of the prominent U wave, theoretically reducing transmural dispersion of repolarization and reducing risk for torsades de pointes. Beta-blockers (including propranolol, atenolol, and sotalol) and sodium channel blockers (including mexiletine, propafenone, and flecainide) have been described as ineffective in select patients [12,31,34]. We have used beta-blockers alone, and in combination with calcium channel blockers with some success, albeit in a modest number of patients.

Aside from preventive measures, an intervention demonstrated to successfully and reliably treat hemodynamically unstable ventricular tachyarrhythmias is the implantable cardioverter-defibrillator (ICD) [35].  The diagnosis of ATS itself can be considered a class IIb indication for ICD placement, citing familial or inherited conditions with a high risk for ventricular tachyarrhythmias as a consideration [36]. A history of cardiac arrest due to ventricular fibrillation or VT would be regarded as a class I indication for ICD placement in ATS, noting that there is evidence and/or general agreement that ICD placement is both useful and effective.  Although sustained VT can also be considered a class I indication for an ICD, many ATS patients are asymptomatic during episodes of sustained ventricular tachycardia [12]. Until further data regarding risk startification becomes available, it seems prudent to reserve ICD therapy for ATS patients with a history of cardiac arrest, syncope, or sustained rapid and/or symptomatic VT.  Furthermore, ICD programming should be tailored to minimize the risk of multiple shocks for VT that may be well tolerated.  Programming no therapy for VT [12], or anti-tachycardia pacing rather than shocks for VT have been advocated [37].

## Conclusions

Andersen-Tawil  syndrome is a rare condition with variable expression, caused by  sporadic mutations or in an autosomal dominant manner.  Mutations in KCNJ2 which encode the inwardly rectifying channel Kir2.1 lead to the clinically apparent phenotype of ventricular arrhythmias, periodic paralysis, and developmental anomalies.  Marked variation in expression confounds ability to diagnose, risk stratify, and effectively treat patients.  ATS differs both on a molecular and phenotypic level from the long QT syndromes, and it has been suggested to abandon its determination as LQT7.  Effective pharmacologic therapy for associated ventricular arrhythmias is lacking, prompting consideration of ICD placement or possibly catheter ablation in high-risk patients.
